# Omeprazole-Associated Atypical Drug Reaction with Eosinophilia and Systemic Symptoms (DRESS) in a Patient with Positive In Vitro Diagnostic Testing to Multiple Proton Pump Inhibitors

**DOI:** 10.1021/acs.chemrestox.4c00225

**Published:** 2024-09-04

**Authors:** Sophie Grice, Sean Hammond, Lucy Hampson, Annette Wagner, Dean J. Naisbitt

**Affiliations:** ‡ Department of Pharmacology and Therapeutics, University of Liverpool, Sherrington Building, Ashton Street, Liverpool L69 3GE, United Kingdom; § ApconiX, Alderley Edge SK10 4TG, U.K.; † Department of Adult Allergy, Guy’s and St Thomas’ Hospital, London SE1 9RT, U.K.

## Abstract

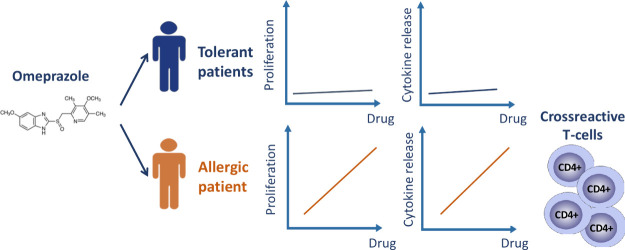

Proton pump inhibitors (PPIs) are a commonly used class of drugs with a good safety profile. However, their use is associated with rare cases of severe skin reaction. Herein, we present details of a patient who developed two episodes of omeprazole-induced delayed-onset hypersensitivity (atypical drug reaction with eosinophilia and systemic symptoms [DRESS]). Lymphocytes from the patient were stimulated to proliferate and secrete cytokines and cytolytic molecules when treated with the drug. T-cell cross-reactivity was observed with structurally related PPIs. Hence, other PPIs have the potential to cause further serious immune-related adverse events in patients who present with hypersensitivity to a primary PPI.

Proton pump inhibitors (PPIs) are frequently prescribed and generally well tolerated drugs for the treatment of acid-related disorders. However, PPIs can be culprit drugs for severe hypersensitivity reactions. Omeprazole is a PPI and prodrug converted to an active sulfenamide form under acidic conditions. The sulfenamide intermediate binds irreversibly to cysteine residues by forming disulfide linkages on parietal cell H+/K+ ATPases (proton pumps). This enables pharmacological inhibition of acid secretion, which has led to indication of omeprazole as a therapeutic in treatment of peptic ulcer disease, gastroesophageal reflux disease, *Helicobacter pylori* associated gastric disease, and Zollinger–Ellison syndrome. The exact frequency of drug hypersensitivity reactions caused by PPIs is unknown. However, there are reports of immediate-type reactions such as anaphylaxis and urticaria/angioedema.^1,2^ In addition, delayed-type reactions such as drug rash with eosinophilia and systemic symptoms (DRESS), and toxic epidermal necrolysis (TEN) have been reported.^3,4^ We present a complex case of a patient who developed angioedema, followed by two episodes of severe cutaneous drug eruption after exposure to a variety of drugs (Figure 1). The first episode is best classified as atypical DRESS,^5,6^ while the second episode presented as exfoliative dermatitis, which may relate to the brevity of treatment and prompt treatment strategy (see details below). The aim of the study was to use investigative laboratory methods with peripheral blood mononuclear cells (PBMCs) from the hypersensitive patient and healthy omeprazole-exposed control subjects to define the culprit drug in the adverse event and to explore the immunogenicity of structurally related PPIs.

**Figure 1:**
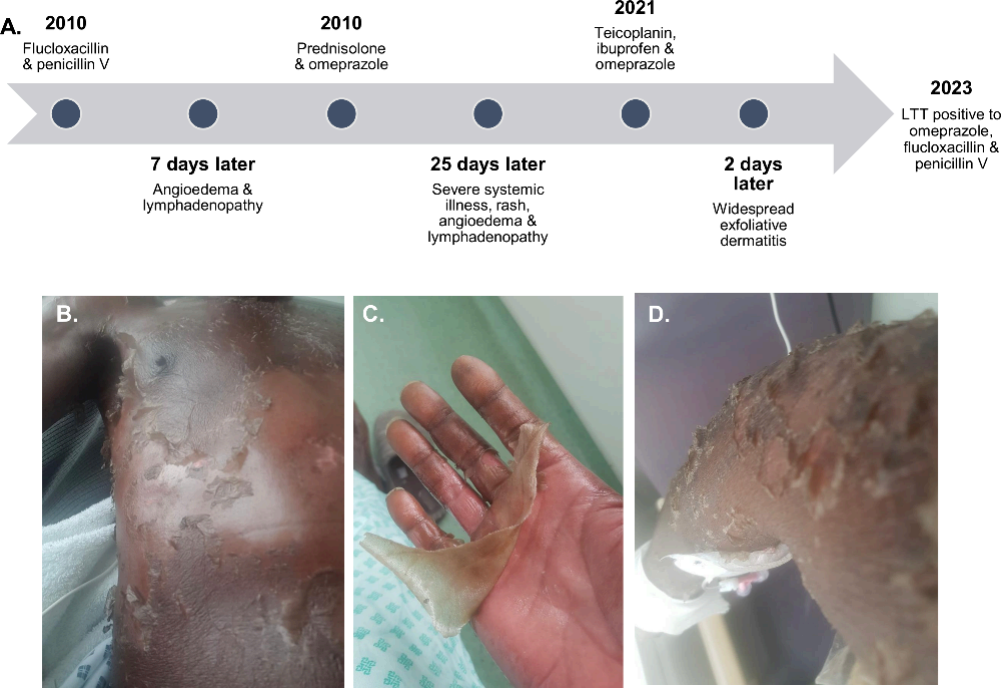
(a) Timeline showing details of adverse events. (b–d) Skin desquamation of patient trunk (b), hands (c) and arm (d).

PBMCs from the patient with hypersensitivity and control subjects were co-cultured with omeprazole (1–50 μM) and other PPIs (esomeprazole, lansoprazole, pantoprazole, and rabeprazole [all 1–25 μM] for 6 days, and lymphocyte proliferation was measured 6 days later through addition of [3H]thymidine for the final 16 h of the experiment. Supernatant was collected prior to [^3^H]thymidine addition for assessment of secreted cytokines using cytometric bead-based immunoassay (LEGENDplex, Biolegend) with an 11-plex panel. IFNγ secretion was also measured using ELISpot after the culture of PBMCs with omeprazole.

The hypersensitive patient was a 62-year-old male with a background of eczema and recurrent staphylococcal folliculitis since childhood. He presented in 2010 to his GP with folliculitis of the scalp. This was treated with flucloxacillin (500 mg tds) for 7 days, with a course of Penicillin V. Three days later he was admitted with facial angioedema and lymphadenopathy. This was interpreted as infection related and he was treated with oral prednisolone (30 mg/day, reducing) and omeprazole (40 mg daily) and discharged a week later. 25 days later he was readmitted with severe systemic illness consisting of widespread rash, facial angioedema and lymphadenopathy (eosinophilia of 4.18 × 109/L, neutrophils 12.4 × 109/L, CRP 27.5 mg/L, IgE > 4000 IU/mL). On admission liver function tests and lymphocyte phenotyping were normal, antistreptolysin O titer was negative, as were EBV, VCA, toxoplasma, and HIV serology. CMV IgG and anti-nuclear antigen (speckled) were positive. Plasma D-Dimer levels were 6930 ng/mL. CT scan revealed widespread lymphadenopathy. Skin biopsy results consistent with a drug eruption are summarized in Table 1. The patient was treated with prednisolone (30 mg reducing) and mycophenolate (500 mg bd) for 1 year under a presumed diagnosis of eczema. In 2012, when the patient was fully recovered, patch testing to standard, steroid, and fragrance series was negative. In 2014, skin prick and intradermal tests to benzylpenicillin, amoxicillin, and flucloxacillin at standard concentrations were negative.

**Table 1:** Clinical Features of Adverse Events and Outcome of Diagnostic Testing

	**Adverse event 1**	**Adverse event 2**	**Adverse event 3**
**Date of event**	2010	2010	2021
**Drug exposure**	Flucloxacillin, penicillin V	Prednisolone, omeprazole	Teicoplanin, ibuprofen and omeprazole
**Time to event**	7 days	25 days	2 days
**Details of reaction**	Angioedema & lymphadenopathy	Widespread rash, facial angioedema and lymphadenopathy	Widespread exfoliative dermatitis
**Biopsy results**	Not performed	Spongiosis, lymphocyte exocytosis, necrotic keratinocytes and eosinophils consistent with drug eruption	Confluent parakeratosis with focal pustule formation. Epidermal dyskeratotic keratinocytes. Superficial and dermal perivascular inflammatory cell infiltrate
**Skin testing**	Not performed	Patch to standard, steroid and fragrance series negative. Prick and intradermal tests to benzylpenicillin, amoxicillin and flucloxacillin negative	Prick and intradermal tests with amoxicillin, teicoplanin and ibuprofen negative
**Lymphocyte transformation test**	Not performed	Not performed	Positive to omeprazole, flucloxacillin and penicillin V

In 2021 the patient was admitted for excision of a Lipoma. Intraoperatively the patient was administered teicoplanin (400 mg). On discharge they received ibuprofen (400 mg tds) and omeprazole (20 mg od) for pain control. Within 48 h the patient developed widespread exfoliative dermatitis, and all medication was stopped (CRP 154 mg/L, no eosinophilia, IgE 108 IU/mL, ALT 75 U/L max, viral serology negative apart from CMV IgG) (Figure 1B–D). Multiple serial sections of a skin biopsy were evaluated, with results summarized in Table 1. The patient was treated with methylprednisolone 500 mg daily for 3 days followed by topical treatment for 4 weeks until recovery. This second episode developed more quickly than the initial atypical DRESS, which is expected given that the immune system is primed to the drug on first exposure and memory cells are reactivated on repeated exposure.

Six months later, skin prick and intradermal tests with amoxicillin, teicoplanin, and ibuprofen were negative on immediate and delayed reading. PBMCs were isolated and used in the lymphocyte transformations test as outlined in Pichler and Tilch^7^ and IFN-γ ELISpot assay carried out according to the manufacturer’s instructions (Mabtech, Sweden). PBMCs were incubated with either media as the negative control, concanavalin A (5 μg/mL) as the positive control or omeprazole (1.6–50 μM), Significant dose-dependent PBMC proliferative responses (up to a stimulation index [SI] of 8), as well as IFN-γ, IL-13, IL-5, granzyme B, perforin and IL-22 secretion were observed in response to omeprazole challenge (Figure 2A–C). PBMCs were also stimulated to proliferate in the presence of penicillin V and flucloxacillin (Figure 2E), which may relate to facial angioedema that developed during the first adverse event episode. In contrast, PBMCs were not activated with amoxicillin (125–4000 μM, piperacillin (62.5–2000 μM), ibuprofen (62.5–2000 μM), teicoplanin (62.5–2000 μM) and paracetamol (62.5–2000 μM).

**Figure 2:**
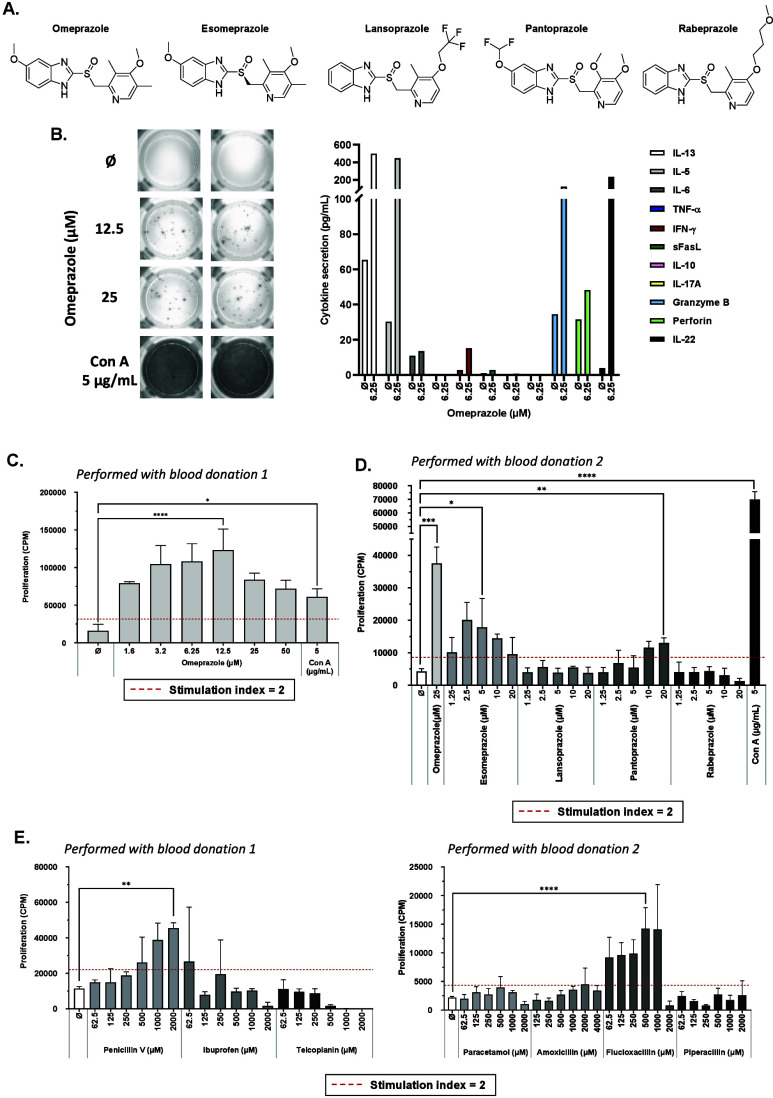
In vitro diagnostic testing. (a) Chemical structures of omeprazole, esomeprazole, lansoprazole, pantoprazole and rabeprazole. (b) Secretion of IFN-γ was visualized via ELISpot assay, and secretion of IL-13, IL-5, IFN-γ, granzyme B, perforin and IL-22 was determined via LEGENDplex assay. (c–e) Lymphocyte transformation test carried out by incubating PBMC with media (negative control) or drugs for 6 days. [^3^H]Thymidine was added for the final 16 h to measure proliferative responses. Significance was determined using one-way ANOVA to compare drug treated wells to media treated wells using Dunnett’s multiple comparisons test (*p < 0.05, **p < 0.01, ***p < 0.001, ****p < 0.0001).

Further experiments were carried out to assess patient cross-reactivity with alternative PPIs that were not administered clinically. Significant PBMC proliferative responses were observed with esomeprazole and pantoprazole compared to media treated wells with SIs of 4 and 3, respectively (Figure 2D). Collectively, these data highlight dual sensitization toward flucloxacillin and omeprazole, with omeprazole identified as the only drug given prior to adverse events 2 and 3. The patient would not agree to challenge with an alternative PPI.

In conclusion, we observed two episodes of LTT that confirmed serious cutaneous reaction in a patient after the administration of omeprazole, indicating PPIs have the capability to cause T-cell-mediated hypersensitivity reactions. Therefore, PPIs should be viewed as potentially causative agents when similar reactions are present in patients. Additionally, in vitro diagnostic testing successfully identified cross-reactivity with other structurally related PPIs. Careful consideration is due when administering alternative PPIs to patients who present with hypersensitivity reactions to a primary PPI, as patients may have T-cells able to cross-react with other PPIs having the potential to cause further serious immune-related adverse events. In future studies, it will be interesting to include this patient in a larger cohort to explore HLA genotypes associated with multiple drug hypersensitivity reactions.

## Abbreviations

PBMC: peripheral blood mononuclear cells
SI: stimulation index
PPIs: proton pump inhibitors.
